# *Post-hoc* Analysis of Outcome of Intravenous Thrombolysis in Infarcts of Infratentorial Localization in the WAKE-UP Trial

**DOI:** 10.3389/fneur.2019.00983

**Published:** 2019-09-11

**Authors:** Ivana Galinovic, Florent Boutitie, Jochen B. Fiebach, Kersten Villringer, Bastian Cheng, Martin Ebinger, Matthias Endres, Jens Fiehler, Ian Ford, Vincent Thijs, Robin Lemmens, Keith W. Muir, Norbert Nighoghossian, Salvador Pedraza, Claus Z. Simonsen, Pascal Roy, Christian Gerloff, Götz Thomalla

**Affiliations:** ^1^Center for Stroke Research Berlin (CSB), Charité-Universitätsmedizin Berlin, Berlin, Germany; ^2^Hospices Civils de Lyon, Service de Biostatistique, Lyon, France; ^3^Université Lyon 1, Villeurbanne, France; ^4^Laboratoire de Biométrie et Biologie Evolutive, Equipe Biostatistique-Santé, Villeurbanne, France; ^5^Klinik und Poliklinik für Neurologie, Kopf- und Neurozentrum, University Medical Center Hamburg-Eppendorf, Hamburg, Germany; ^6^Neurologie der Rehaklinik Medical Park Humboldtmühle, Berlin, Germany; ^7^Klinik und Hochschulambulanz für Neurologie, Charité-Universitätsmedizin Berlin, Berlin, Germany; ^8^German Center for Neurodegenerative Diseases Within the Helmholtz Association, Partner Site Berlin, Bonn, Germany; ^9^Department of Diagnostic and Interventional Neuroradiology, University Medical Center Hamburg-Eppendorf, Hamburg, Germany; ^10^Robertson Centre for Biostatistics, University of Glasgow, Glasgow, United Kingdom; ^11^Stroke Theme, Florey Institute of Neuroscience and Mental Health, University of Melbourne, Heidelberg, VIC, Australia; ^12^Austin Health, Department of Neurology, Heidelberg, VIC, Australia; ^13^Department of Neurology, University Hospitals Leuven, Leuven, Belgium; ^14^Department of Neurosciences, Experimental Neurology, KU Leuven–University of Leuven, Leuven, Belgium; ^15^VIB, Laboratory of Neurobiology, Center for Brain and Disease Research, Leuven, Belgium; ^16^Institute of Neuroscience and Psychology, University of Glasgow, Glasgow, United Kingdom; ^17^Department of Stroke Medicine, Université Claude Bernard Lyon 1, Lyon, France; ^18^Department of Radiology, Hospital Dr. Josep Trueta, Institut de Diagnostic per la Image (IDI), Institut d'Investigació Biomèdica de Girona (IDIBGI), Girona, Spain; ^19^Department of Neurology, Aarhus University Hospital, Aarhus, Denmark

**Keywords:** infratentorial infarct, infratentorial stroke, intravenous thrombolysis, alteplase, MRI, WAKE-UP

## Abstract

**Introduction:** In WAKE-UP (Efficacy and Safety of MRI-based Thrombolysis in Wake-Up Stroke), patients with an acute stroke of unknown onset time were randomized to treatment with intravenous alteplase or placebo, guided by MRI.

**Methods:** In this exploratory *post-hoc* secondary analysis we compared clinical and imaging data, as well as treatment effects and safety of intravenous thrombolysis between patients with infra- vs. supratentorial stroke.

**Results:** Forty-eight out of 503 randomized patients (9.5%) presented with a stroke involving the cerebellum or brainstem. Patients with infratentorial stroke were younger compared to patients with supratentorial stroke (mean age 60 vs. 66 years), more frequently male (85 vs. 62%), and less severely affected (median NIHSS 4.5 vs. 6.0). There was no heterogeneity for treatment effect between supratentorial (OR 1.67 95% CI 1.11–2.51) and infratentorial (OR 1.31 95% CI 0.41–4.22) sub-groups (test for interaction *p* = 0.70). In patients with infratentorial stroke, favorable outcome [a score of 0–1 on the modified Rankin scale (mRS) at 90 days] was observed in 12/22 patients (54.5%) in the alteplase group and in 13/25 patients (52.0%) in the placebo group (*p* = 0.59). The primary safety endpoint (death or mRS 4–6 at day 90) occurred in three patients of the alteplase group (13.6%) and three patients in the placebo group (12.0%); *p* = 0.74.

**Discussion:** WAKE-UP was underpowered for demonstrating treatment effect in subgroup analyses however, based on our current results, there is no evidence to recommend withholding MRI-guided thrombolysis in patients with unknown onset stroke of infratentorial localization.

## Introduction

The WAKE-UP trial (a randomized, double-blind, placebo-controlled trial, ClinicalTrials.gov number, NCT01525290) (1) was an investigator-initiated, multicenter, randomized, double-blind, placebo-controlled clinical trial which provided evidence of clinical benefit of MRI-guided treatment with intravenous alteplase in acute stroke patients with an unknown time of symptom onset. The study was based on the concept of DWI-FLAIR mismatch, with lesions visible on diffusion-weighted imaging (DWI) but not clearly visible on fluid-attenuated-inversion-recovery (FLAIR) identifying patients within 4.5 h of stroke onset. This concept was established through previous studies reporting a high specificity (78%) and positive predictive value (83%) of the DWI-FLAIR mismatch in identifying hyperacute stroke patients (2). Since previous studies have shown that FLAIR signal changes might develop more slowly in infratentorial than in supratentorial stroke (3, 4), there remained uncertainty about the safety and efficacy of thrombolysis based on DWI-FLAIR mismatch in this cohort. In WAKE-UP, patients were randomized irrespective of the localization of the acute ischemic stroke, providing us with an opportunity to perform a subgroup analysis of patients with brainstem and cerebellar strokes. The objective of the current study was to investigate the safety and efficacy of intravenous alteplase administered based on the presence of a DWI-FLAIR mismatch in patients with infratentorial strokes.

## Materials and Methods

### Study Design

The national competent authorities and ethics committees in all participating countries approved the study. WAKE-UP was registered at https://www.clinicaltrials.gov. Unique identifier: NCT01525290. URL: https://www.clinicaltrialsregister.eu. Unique identifier: 2011-005906-32. Informed consent was obtained from all patients prior to enrollment into the trial. Patients were included in this substudy if the sole location of their acute ischemic lesion (based on baseline DWI) was in one or more of the following brain regions: the pons, medulla oblongata, cerebellum, or mesencephalon. We examined demographic characteristics, clinical, and imaging data at baseline and follow-ups for this subgroup of patients and compared them to patients with a supratentorial stroke.

### Outcome Measures

The primary efficacy endpoint was favorable outcome defined as a score of 0–1 on the modified Rankin scale (mRS) at final follow up (90 days post-stroke). As a secondary efficacy endpoint we evaluated an ordinal analysis of the mRS (“shift analysis”). The primary safety endpoint was death or dependence (defined as a score of 4–6 on the mRS at 90 days post-stroke), additional safety outcomes were the incidence of symptomatic intracerebral hemorrhage (SICH) according to the protocols of SITS-MOST, ECASS II, ECASS III, NINDS, and parenchymal hemorrhage type 2 (PH-2) on follow-up imaging 22–36 h after treatment (5–8).

### Statistical Analysis

Statistical analyses of treatment effects were performed in the intention-to-treat population. To investigate the interaction between stroke location (i.e., infra- vs. supratentorial lesion) and treatment effect on the primary endpoint, we used an unconditional logistic regression model, relating the log-odds of the primary outcome with the covariate of interest, the treatment group, and their interaction. The interaction term was tested with the Wald-Chi-squared test, and the treatment effect (odds ratio [OR]) and its 95% confidence interval (CI) was estimated for each category of the stroke location. We furthermore repeated the analysis of primary and secondary endpoints as in the original trial analysis in the subpopulation of patients with infratentorial strokes. The main efficacy variable as well as the safety endpoints were assessed using an unconditional logistic regression analysis, fitted to estimate the OR and its 95% CI interval. The categorical shift in the distribution of mRS scores was analyzed by fitting a proportional-odds logistic regression model. All analyses were adjusted for the stratification parameters age and NIHSS. All tests were carried out with a two-sided alpha level of 5% without correction for multiple comparisons.

## Results

### Comparison of Infra- vs. Supratentorial Strokes in the Screened Population

Of 1,362 patients screened for WAKE-UP, 84 (6%) had a cerebellar and/or brainstem stroke. These patients were younger (mean 62.5 years, SD 12.0) when compared to patients with supratentorial strokes (mean 65.3 years, SD 11.8; *p* = 0.02), and were more often male (64/84, 76% as opposed to 769/1,278, 60%; *p* = 0.004). In addition, the NIHSS score at baseline (median; IQR) was lower in patients with infratentorial stroke (5; 3–6) than in patients with supratentorial stroke (6; 4–11), *p* < 0.001. We did not identify a difference in cardiovascular risk factors e.g., arterial hypertension, diabetes mellitus, hypercholesterolemia, or history of ischemic stroke between groups. However, there was a higher prevalence of atrial fibrillation in patients with supratentorial strokes (106/1,278, 8%) as compared to patients with infratentorial strokes (1/84, 1%; *p* = 0.03). In infratentorial stroke patients, there were numerically fewer FLAIR positive lesions compared to the group with a supratentorial stroke localization (23/84, 27% as opposed to 479/1,278, 38%; *p* = 0.09).

### Comparison of Infra- vs. Supratentorial Strokes in the Intention-to-Treat Population

**Figure 1:**
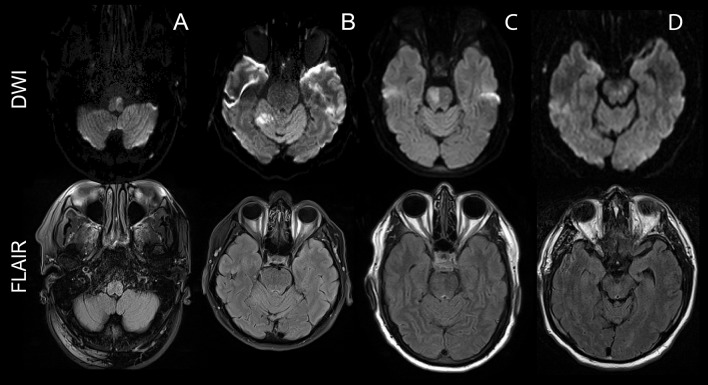
Examples of infratentorial strokes in the intention-to-treat WAKE-UP cohort. The upper row shows diffusion-weighted images depicting the acute ischemic stroke. The bottom row shows a FLAIR image of the corresponding slice, depicting a lack of signal hyperintensity in the area of the acute stroke. The different columns offer examples for the different stroke locations included into this substudy: **(A)** depicts a stroke of the ventral left portion of the medulla oblongata, **(B)** a right-sided cerebellar stroke in the feeding territory of the superior cerebellar artery, **(C)** a right-sided stroke in the pons, and **(D)** a focal mesencephalic stroke.

Of the 503 patients who were randomized into the trial, 48 (9.5%) presented with a stroke in an infratentorial brain region. Twenty-six patients (54%) were assigned to placebo with one patient not having received infusion and 22 patients received alteplase (46%). Twenty-eight patients had an ischemic lesion in the pons (58%), nine patients had cerebellar stroke (19%), seven patients presented with an infarct in the medulla oblongata (15%), two patients had a stroke in the mesencephalon (4%), and two patients (4%) had strokes in more than one location (brainstem plus cerebellum); see Figure 1 for examples. The distribution of lesion localization was extremely uniform between the placebo and the alteplase group. As in the overall screened population, patients randomized with infratentorial strokes were also younger, more often male and less severely affected at admission to hospital than those with supratentorial strokes (Table 1). However, at day 7 post-stroke, there was no longer a difference in the NIHSS scores between the groups (Table 1). As expected, the median baseline volume of infratentorial strokes was smaller than that of supratentorial strokes (0.8 vs. 2.6 ml; *p* < 0.001). There was no statistically significant difference in the percentage of patients reaching the primary efficacy endpoint in infratentorial stroke (25/47, 53%) vs. supratentorial stroke (208/443, 47%); *p* = 0.45. In addition, the rate of reaching the primary safety endpoint did not differ, with 6/47 (13%) patients in the infratentorial group and 72/443 (16%) patients in the supratentorial group (*p* = 0.23). There were no symptomatic intracerebral hemorrhages in the infratentorial patient group and only three patients experienced petechial hemorrhagic transformation (HI-1 and HI-2) (6%, as compared to 73/455 or 16% in the supratentorial group).

**Table 1:** Group comparison between patients with an infra- and a supratentorial localization of the acute stroke in the intention-to-treat population.

	**Supratentorial stroke *N* = 455**	**Infratentorial stroke *N* = 48**	
Mean age (years) (SD)	65.8 (11.3)	59.9 (12.2)	*p* < 0.001
Gender (male), *N* (%)	284 (62.4%)	41 (85.4%)	*p* = 0.001
Median symptom recognition to start of treatment (hours) (IQR)	3.1 (2.5–3.9)	3.2 (2.7–3.9)	*p* = 0.227
Arterial hypertension, *N* (%)	241 (53.0%)	25 (52.1%)	*p* = 0.912
Diabetes mellitus, *N* (%)	72 (15.8%)	10 (20.8%)	*p* = 0.680
Atrial fibrillation, *N* (%)	58 (12.8%)	1 (2.1%)	*p* = 0.049
Hypercholesterolemia, *N* (%)	160 (35.2%)	18 (37.2%)	*p* = 0.387
Median NIHSS at baseline (IQR)	6.0 (4.0–10.0)	4.5 (3.0–6.0)	*p* = 0.001
Median NIHSS at 7 days post-stroke (IQR)	2.0 (1.0–6.0)	2.0 (0.0–6.0)	*p* = 0.156
Median stroke volume at baseline (ml) (IQR)	2.6 (0.9–9.6)	0.8 (0.3–1.8)	*p* < 0.001
Median stroke volume at follow up (ml) (IQR)	3.5 (1.1–19.5)	0.8 (0.3–3.1)	*p* < 0.001

*Follow up was 22–36 h after treatment*.

**Figure 2:**
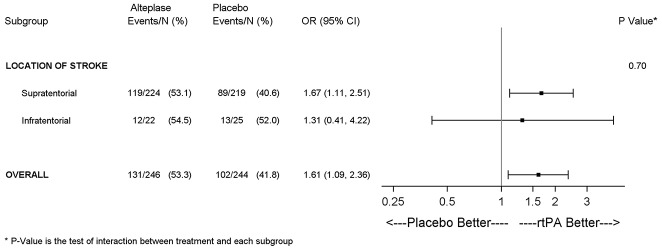
Forest plots depicting the effect (adjusted OR) of alteplase on favorable outcome in patients with a supra- and infratentorial stroke with no evidence of a significant interaction between stroke localization and treatment effect.

Treatment with alteplase was associated with higher odds of favorable outcome with no significant heterogeneity of treatment effect for stroke subtype (infratentorial vs. supratentorial). The adjusted OR for favorable outcome with alteplase was 1.31 (95% CI 0.41–4.22) in patients with infratentorial infarct and 1.67 (95% CI 1.11–2.51) in patients with supratentorial infarct (test for interaction, *p* = 0.70; see Figure 2).

### Results in the Subpopulation of Infratentorial Strokes

Baseline parameters were comparable for patients randomized to receiving alteplase or placebo (Table 2). Favorable outcome was observed in 12 out of 22 patients (55%) in the alteplase group and in 13 out of 25 patients (52%) in the placebo group (adjusted OR, 1.38; 95% CI 0.42–4.56; *p* = 0.60). The 90 day distributions of mRS scores for the remaining categories were, for the alteplase and the placebo arm, respectively, five patients (23%) vs. seven patients (28%) with mRS of 2, two patients each (9 vs. 8%) for mRS of 3, two patients (9%) vs. one patient (4%) for mRS of 4 and one patient (5%) vs. two patients (8%) with an mRS of 5 or 6. We were unable to show a trend for a shift toward better outcomes in those infratentorial stroke patients treated with alteplase as compared to those who received placebo (adjusted common OR 1.19; 95% CI 0.41–3.42; *p* = 0.75). There were no SICH or deaths in the group of patients with infratentorial strokes, regardless of the administered treatment. The primary safety endpoint (death or mRS score 4–6 at day 90) occurred in three patients of the alteplase group (14%) and three patients in the placebo group (12%); *p* = 0.74. Petechial hemorrhagic transformation (HI-1 and HI-2) occurred in two patients (9%) who have received alteplase and one patient (4%) who received placebo (*p* = 0.59).

**Table 2:** Comparison between patients who received alteplase and those who received placebo in the group of patients with an infratentorial stroke localization.

	**Alteplase *N* = 22**	**Placebo *N* = 26**	
Mean age (years) (SD)	62.6 (10.3)	57.7 (13.5)	*p* = 0.230
Gender (male), *N* (%)	20 (90.9%)	21 (80.8%)	*p* = 0.429
Median symptom recognition to start of treatment (hours) (IQR)	3.1 (2.6–3.6)	3.5 (2.9–4.0)	*p* = 0.272
Arterial hypertension, *N* (%)	12 (54.6%)	13 (50.0%)	*p* = 0.780
Diabetes mellitus, *N* (%)	7 (31.8%)	3 (11.5%)	*p* = 0.152
Atrial fibrillation, *N* (%)	1 (4.6%)	0 (0.0%)	*p* = 0.205
Hypercholesterolemia, *N* (%)	11 (50.0%)	7 (26.9%)	*p* = 0.138
Median NIHSS at baseline (IQR)	5.0 (3.0–8.0)	4.0 (3.0–5.0)	*p* = 0.295
Median NIHSS at 7 days post-stroke (IQR)	1.5 (0.0–6.0)	2.0 (1.0–4.0)	*p* = 0.580
Median stroke volume at baseline (ml) (IQR)	0.8 (0.2–1.9)	0.7 (0.3–1.3)	*p* = 0.820
Median stroke volume at follow up (ml) (IQR)	0.8 (0.3–3.1)	0.9 (0.4–3.1)	*p* = 0.656

*Follow up was 22–36 h after treatment*.

## Discussion

WAKE-UP demonstrated a clear clinical benefit of treatment with intravenous alteplase in patients with an acute ischemic lesion visible on DWI but not yet evidently visible on FLAIR imaging. In this secondary *post hoc* analysis, we focused on the treatment effect and further elucidated the clinical characteristics and outcome in a subpopulation of WAKE-UP patients with a brainstem or cerebellar DWI lesion. We did not observe heterogeneity of the treatment effect based on stroke localization. There was no difference in death or dependence between the two treatment arms and no symptomatic intracerebral hemorrhages occurred in our cohort of patients with infratentorial ischemic lesions. Thus, we do not recommend excluding patients with infratentorial stroke of unknown time of symptom onset with a DWI/FLAIR mismatch from intravenous thrombolysis. However, the analysis was unable to prove benefit of thrombolysis in the alteplase arm, presumably due to the fact that the trial was underpowered to demonstrate superiority in this context. An additional limitation of the study was the inclusion of patients with relatively mild strokes, making the generalization of the findings on patients suffering severe stroke difficult.

There were some clinical differences between the subgroups. Infratentorial stroke patients were younger and more frequently male as compared to patients with supratentorial stroke, which corresponds to trends reported in literature (4, 9). They also had lower baseline NIHSS scores and smaller stroke volumes, results which are logical and equally in line with previous observations (4, 9). The higher prevalence of atrial fibrillation in patients with supratentorial as opposed to infratentorial stroke, as found in our study, has similarly been previously reported (9). These findings could in part be explained by the younger age and male predominance in our cohort, as there is a known higher prevalence of atrial fibrillation in older women leading to an increased risk of severe cardioembolic strokes in the anterior circulation in females (10).

In the context of stroke of unknown onset, previous research has shown that ischemic lesions in infratentorial brain regions likely take longer to develop a FLAIR hyperintense signal (4) which subsequently implies that a proportion of patients treated based on the presence of a DWI-FLAIR mismatch are likely beyond the conventional 4.5 h time window for rt-PA. Justifiably, at the time of the study's conduct, this raised potential safety concerns. Our current analysis suggests that these concerns were unfounded as no deaths and no parenchymal hemorrhages (symptomatic or otherwise) occurred in our subgroup of patients with infratentorial strokes. This may in part be the effect of the mild stroke severity in our cohort, but is also in line with previous large cohort studies which have reported very low SICH rates in patients with isolated brainstem and cerebellar strokes (9, 11). Also befitting the literature (9, 11), the percentage of observed hemorrhagic transformations was lower in the subgroup of infratentorial as compared to supratentorial stroke patients, which comes to no surprise as it is often not associated with thrombolysis but rather dependent on stroke size and severity (12). This underlines the safety of patient selection for intravenous thrombolysis based on the DWI-FLAIR mismatch approach in infratentorial, mild to moderate stroke. Some previous studies have pointed to a lesser importance of the time to treatment (with regards to developing intracranial hemorrhage or unfavorable outcome) in posterior circulation strokes as compared to anterior circulation strokes (13). However, it is also conceivable that the stage of tissue damage depicted by a positive DWI but negative FLAIR signifies a condition in which thrombolysis is still safe, irrespective of the actual time which elapsed since the onset of ischemia. Other recent studies have equally pointed to the safety (as well as efficacy) of acute stroke treatment in the unknown and extended time window in carefully selected patient cohorts, further moving evidence away from time-based and toward tissue-based models and individually tailored therapy (14).

Our analysis did not show a difference between rt-PA and placebo on the treatment effect in patients with unknown onset stroke of infratentorial localization. There is a general lack of information on this subject in the literature, as very few randomized, controlled trials or phase IV studies evaluating the safety and efficacy of iv tPA in posterior circulation strokes are available (15, 16). Hence our findings, notwithstanding the small cohort size, represent knowledge novel and relevant to the field. Within the WAKE-UP study population itself, the percentage of infratentorial alteplase treated patients who reached favorable outcome was slightly higher than in the overall study cohort (54.5 vs. 53.3%) but the placebo group of infratentorial patients did remarkably well with 52% reaching favorable outcome (as opposed to only 41.8% of patients in the complete WAKE-UP population). This is not surprising as some preexisting studies have shown that up to 60% of patients with a stroke in the posterior circulation recover to the point of being able to carry out all usual duties and activities despite lack of treatment (16). This same study (16) identified a cutoff baseline NIHSS score of 5 or below as one with a high sensitivity and specificity for predicting favorable outcome in untreated patients with a posterior circulation stroke (and 75% of the placebo cohort in our current substudy had a baseline NIHSS of 5 or less). Hence, a high response rate in the placebo group of our study was to be expected, a fact which has arguably undermined the potential to detect treatment effect in some other previously conducted trials (17).

## Data Availability

The datasets generated for this study are available on request to the corresponding author.

## Ethics Statement

The studies involving human participants were reviewed and approved by the national competent authorities and ethics committees in all participating countries approved the study. The patients/participants provided their written informed consent to participate in this study.

## Author Contributions

IG: literature research, conception and design of the study, and manuscript drafting. FB: statistical analysis. IG, JBF, KV, BC, MEb, MEn, JF, IF, VT, RL, KM, NN, SP, CS, PR, CG, and GT: protocol development of the WAKE-UP study and patient recruitment. GT: ethical approval. All authors reviewed, edited, and approved the final manuscript.

### Conflict of Interest Statement

FB reports grants from University Medical Center Hamburg-Eppendorf during the conduct of the study. CG, GT, IG, JBF, KM, MEb, MEn, SP, and VT report grants from European Union Seventh Framework Program during the conduct of the study. The remaining authors declare that the research was conducted in the absence of any commercial or financial relationships that could be construed as a potential conflict of interest.
